# Morphologic Measurements of Anatomic Landmarks in Pulp Chambers of Human First Molars: A Study of Bitewing Radiographs

**Published:** 2008-01-10

**Authors:** Leila Khojastepour, Naser Rahimizadeh, Akbar Khayat

**Affiliations:** 1 *Department of Oral Radiology, Dental School, Shiraz University of Medical sciences, Shiraz, Iran*; 2 *Endodontist, Shiraz, Iran*; 3 *Department of Endodontics, Dental School, Shiraz University of Medical sciences, and Iranian Center for Endodontic Research, Tehran, Iran*

**Keywords:** Bitewing, Dental Pulp Cavity, Molar, Morphology, Radiography

## Abstract

**INTRODUCTION:** Knowledge of the anatomic location and dimension of the molar pulp chamber may preserve healthy pulp during operative procedure and reduce risk of perforation of chamber during access preparation. A review of literatures regarding the morphology of pulp chamber however revealed very little information; so the aim of this *in vivo *study was to measure the dimensions of first molar pulp chambers as seen on bitewing (BW) radiographs.

**MATERIALS AND METHODS:** In this cross-sectional study molar's BW radiographs of 130 subjects in two age groups were taken under standardized conditions (group of 18-25 years old subjects and group of 50-65 years old subjects). The films were digitized and nine anatomical landmarks were evaluated from the image of each first molar as follow: A; mesial to distal pulp horn distance, B; mesial to distal walls at the middle of pulp chamber, C; mesial to distal orifices, D; mesial cusp tip to its horn, E; distal cusp tip to its horn, F; pulp chamber height, G; pulp chamber floor to furcation, H; pulp chamber ceiling to furcation, I; cusp tips to furcation. The data were evaluated by using AUTOCAD (2007) software with 0.00 precision. Two way ANOVA test (Uni-variant analysis) were used to determine the interaction between restoration and age on pulp chamber dimensions.

**RESULTS:** There was no significant difference in pulp chamber dimensions between the genders. Although there was significant reduction in the size of pulp chamber with advancing age, no significant differences were found in the restored and non restored teeth.

**CONCLUSION:** The finding of this study showed size reduction and changes of pulp chamber with age so may enhance knowledge to minimize errors during endodontics treatments.

## INTRODUCTION

The preservation of healthy pulp during operative procedure and successful manage- ment in case of disease are two of the most important challenges for a clinical dentist (1). One of the complications of endodontics is perforation into the furcation area during access preparation of molar teeth which often result in extraction of the tooth (2). Access preparations are performed by a qualitative method involving the clinician’s tactile perception and knowledge of dental anatomy. However, a reliance on tactile perception alone may lead to undesirable results, including perforation in the floor of the pulp chamber (3). A review of literature regarding the morphology of the pulp chamber revealed very little information. One study measured the distance from the floor of the pulp chamber to five predetermined sites on the furcation root surface and found it to range from 2.7-3 mm for both mandibular and maxillary molars (4). Another study reported that the mean distance from the pulp chamber floor to the point of root separation of maxillary molars was equal to or less than 3 mm in 86% of the teeth measured (5).

Accidental pulp exposure could be minimized if we have detailed knowledge of anatomy and common morphological variations in molar teeth. Obviously, a precise knowledge of the location and dimension of molar pulp chamber may reduce perforation of the floor of the chamber during the access preparation.

**Figure 1:**
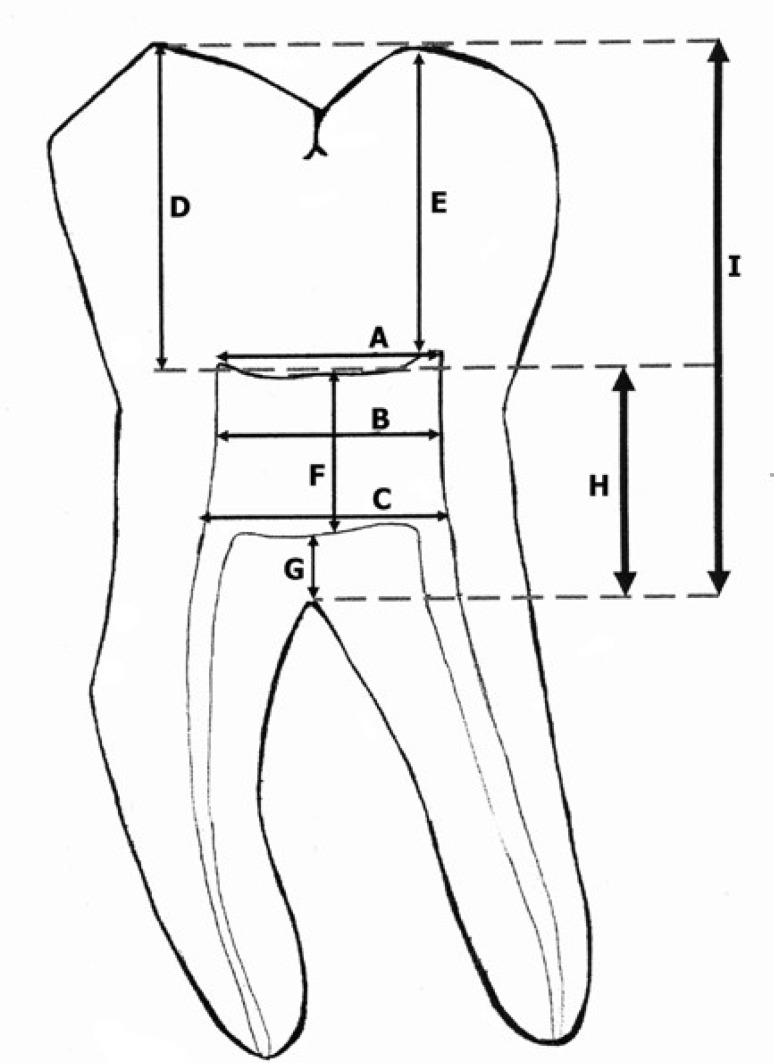
Location of measurements for mandibular molars mesial to distal pulp horn (A), mesial to distal walls at the middle of pulp chamber (B), mesial to distal orifices (C), mesial cusp tip to its horn (D), distal cusp tip to its horn (E), pulp chamber height (F), pulp chamber floor to furcation (G), pulp chamber ceiling to furcation (H), cusp tips to furcation (I).

Dental practitioners rely on BW radiographs as a routine diagnostic tool, supplementing these with periapical views for endodontic diagnosis and treatment. Considering projection geo- metry, the bitewing radiographs is said to provide the optimal view of the pulp chamber and may help to avoid perforation of molars during endodontic access cavity preparation (6,7). However, like all radiographic images, their limitation is the two-dimensional image of a three-dimensional object.

The aim of present study was to investigate the dimensions of first molar pulp chambers as seen on a large collection of bitewings from individuals in two groups with different mean age and relate these to the mean age of the patients as well as the presence/or absence of restorations.

## MATERIALS AND METHODS

Bitewing radiographs were taken from two groups of volunteer that were referred to the radiology department of Shiraz Dental School.

A total of 260 bitewing films from 130 individuals from two age groups were included in this investigation. Group of 18-25 years old consist of 62 subjects with the mean age of 22.8, and group of 50-65 years old consist of 68 subjects with the mean age of 56.2. Ethical approval was granted by the university, and the consent of the subjects was gained to access their radiographs. The consent form requested details such as age and sex. The films were exposed using the same X-ray machine (Intra Planmeca, Finland 70 KVP, 10 mA) with Agfa Ektaspeed film supported in a Rinn bitewing holder & beam alignment device (Dentsply Rinn, UK). A piece of 5mm orthodontic wire (0.7mm) was placed on radiographic films for film calibration and magnification adjustment. Processing was automatic (Peripro Air Technique machine, USA), using Teyph-Saz processing solutions. All the radiographs (130 individuals- 260 bitewing) were coded and then digitally scanned using a Microtech I 800 scanners. The teeth with crowns or restoration that obscured image of pulp chambers were excluded from study. Considering exclusion criteria, a total of 402 maxillary and mandibular first molars were included (234 maxillary and 168 mandibular molars). Nine selected anatomical landmarks of pulp chambers were measured and analysed using AUTOCAD (2007) program with 0.00 precision after calibration by an operator who was unaware of the patient details.

A drawing of the location of measurements for maxillary and mandibular molars is shown in Figure 1. Nine direct measurements were taken of each tooth as follow: mesial to distal pulp horn (A), mesial to distal walls at the middle of pulp chamber (B), mesial to distal orifices (C), mesial cusp tip to its horn (D), distal cusp tip to its horn (E), pulp chamber height (F), pulp chamber floor to furcation (G), pulp chamber ceiling to furcation (H), cusp tips to furcation (I). The presence and location of restorations were recorded.

**Table 1:** Effects of age and restoration on Mean ± SD of different measuring dimensions (A to I) of maxillary first molars

**V** **ar** **i** **a** **bl** **e** **s**	**Age**		P Value	**R** **e** **st** **o** **r** **a** **ti** **o** **n**		P Value	**A** **g** **e R** **e** **st** **o** **r** **a** **ti** **o** **n**
**Measuring**	n=114	N=120		n=124	n=110		P Value ^a^
**D** **i** **m** **e** **n** **s** **io** **ns**	18-25 y/o	50-65 y/o		Non Restored	Restored		
A	3.88±0.83	3.00±0.72	0.000^a^	3.61±1.14	3.30±.87	0.064	0.836
B	3.29±0.86	2.79±0.69	0.001^a^	3.14±0.78	2.92±0.82	0.112	0.501
C	2.79±0.91	2.65±0.85	0.514	2.83±0.91	2.65±0.87	0.298	0.164
D	5.45±0.91	6.19±0.79	0.000^ a^	5.67±0.99	5.85±1.10	0.325	0.060
E	5.90±0.89	6.46±0.72	0.000^ a^	6.11±0.84	6.24±0.83	0.365	0.079
F	3.19±0.54	2.11±0.52	0.000^ a^	2.65±0.81	2.63±0.70	0.789	0.639
G	2.86±0.57	3.51±0.49	0.000^ a^	3.22±0.70	3.20±0.52	0.936	0.094
H	6.02±0.84	5.92±0.81	0.008^ a^	5.78±0.90	5.81±0.81	0.892	0.274
I	11.54±1.20	11.94±0.96	0.259	11.48±1.65	11.66±1.7	0.509	0.243

*a:* *Significant* *p* *value* *<* *0.05,* *mesial* *to* *distal* *pulp* *horn* *(A),* *mesial* *to* *distal* *walls* *at* *the* *middle* *of* *pulp* *chamber* *(B),* *mesial to* *distal orifices* *(C),* *mesial* *cusp* *tip* *to* *its* *horn* *(D),* *distal* *cusp* *tip* *to* *its horn* *(E),* *pulp* *chamber* *height* *(F), pulp* *chamber floor* *to* *furcation* *(G), pulp* *chamber ceiling* *to* *furcation* *(H),* *cusp* *tips to* *furcation* *(I)*

**Table 2:** Effects of age and restoration on Mean ± SD of different measuring dimensions (A to I) of mandibular first molars

**V** **ar** **i** **a** **bl** **e** **s**	**Age**		P Value	**R** **e** **st** **o** **r** **a** **ti** **o** **n**		P Value	**A** **g** **e R** **e** **st** **o** **r** **a** **ti** **o** **n**
**Measuring**	n=108	n=60		n=86	n=82		P Value ^a^
**D** **i** **m** **e** **n** **s** **io** **ns**	18-25 y/o	50-65 y/o		Non Restored	Restored		
A	4.44±0.59	3.74±0.55	0.000^ a^	4.27±0.64	4.08±0.68	0.077	0.716
B	4.10±0.69	3.61±0.68	0.000^ a^	4.04±0.71	3.97±0.75	0.053	0.517
C	3.90±0.69	3.50±0.71	0.008^ a^	3.78±0.74	3.71±0.72	0.466	0.792
D	5.10±0.88	5.65±0.71	0.002^ a^	5.31±0.69	5.33±1.08	0.640	0.750
E	5.41±0.96	5.64±0.84	0.139	5.48±1.02	5.50±1.01	0.857	0.938
F	2.32±0.56	1.65±0.50	0.000^ a^	2.20±1.03	2.03±0.49	0.239	0.644
G	2.89±0.61	3.31±0.43	0.049^ a^	3.03±0.57	2.92±0.54	0.485	0.985
H	5.22±0.85	4.78±0.71	0.026^ a^	5.16±0.43	4.90±0.70	0.149	0.763
I	10.41±1.27	10.45±1.03	0.546	10.51±1.07	10.25±1.62	0.529	0.642

*a:* *Significant* *p* *value* *<* *0.05;* *mesial* *to* *distal* *pulp* *horn* *(A);* *mesial* *to* *distal* *walls* *at* *the* *middle* *of* *pulp* *chamber* *(B);* *mesial to* *distal orifices* *(C);* *mesial* *cusp* *tip* *to* *its* *horn* *(D);* *distal* *cusp* *tip* *to* *its horn* *(E);* *pulp* *chamber* *height* *(F); pulp* *chamber floor* *to* *furcation* *(G); pulp* *chamber ceiling* *to* *furcation* *(H);* *cusp* *tips to* *furcation* *(I)*

Reliability of measurements was checked in pilot study involving linear measurements from radiographs according to the calibration of orthodontic wire, revealing a high correlation of repeated measurements (*r *= 0.90).

Statistical significance of the results was determined using Two way ANOVA analysis.

## RESULTS

Hundred and twenty-four bitewing radiographs in 18-25 and 136 in 50-65 year old group were available. Hundred and eighteen first molars were excluded because of being extracted, root filled, crowned, or had images on which some measurements were not possible; so 402 teeth were analysed out of 520. There were 234 maxillary first molars and 168 mandibular first molars. Restorations were present in 47% of maxillary first molars and 48.8% of mandibular first molars.

There was no significant difference among the means and standard deviations (SD) of the nine measurements of the molars in terms of group and gender (Student’s T test p>0.05); so the data were recorded and analysed together. Means and SD of nine measurements (A to I) made per tooth of both groups are shown in Table 1 and Table 2 for maxillary and mandibular first molars, respectively. Two way ANOVA analysis has shown that there is no significant interaction (P>0.05) between restoration and pulp chamber dimension as both restored and non restored teeth have shown the same process of reduction in pulp chamber dimension with age. In addition, considering the age factor there is no significant difference among measuring dimensions of restored and non restored maxillary first molars, as well as mandibular first molars (P>0.05). Based on available data however, most of the recorded means of the pulp chambers dimension for both maxillary and mandibular first molar were significantly lower in older individual in presence of restoration.

## DISCUSSION

Bitewing technique has been applied to detect the anatomic landmarks of upper and lower molar pulp chamber because of its reliability (8), and it was used by other investigators (9-12). Philippas was the first whose study gave quantitative data on pulp chambers size in a radiological investigation (13). Chandler *et al*. studied coronal pulp dimension in human first molar teeth using bitewing radiographs and pointing out that pulp chamber morphology seen better on these films than periapical radiographs (9). Their study was more comprehensive comparing to previous studies that excluded molars (10), did not include first molars (12), and featured fewer than 10 teeth (14). On the other hand, Kandemir (12) studied the radiographic determinability of the distance between the pulp horns in the permanent first and second molar teeth, and he found more reliable identification of pulp location in mandibular molar than in maxillary molars using bitewings. This was considered to be partly a matter of beam angulation and partly because of the fact that the pulp horns are more visible and longer at the lingual side of mandibular molars .In maxillary molars, the mesio-buccal horn is longer and more visible.

In this *in vivo *study we also used bitewing radiograph for measuring nine desired dimensions. This technique has either few or no geometric distortion, or it used widely for clinical detection of interproximal caries.

Sexual dimorphism of teeth has been studied by means of odontometric analyses, based on data of the mesio-distal and bucco-lingual crown diameters in permanent dentition (15,16). In general, male teeth have been found to be larger than those of the female (most pronounced in the canine tooth) and an excellent review on the subject has been published by Kieser (17). However, there is no sufficient research on pulp chamber dimension that could prove sexual dimorphism and the few presence researches suggest different results (9,18,19). In the present study, we found no significant difference in pulp chamber dimensions between the genders. This is in contrast to Chandler *et al. *(9) and in accordance with Moss (18) as well as Shaw and Jones (19). Moss (18) considered that neither the size nor the shape of the human pulpal outline was related to gender. Shaw and Jones also found no significant differences between width of the pulp chamber of girls and boys when they examined the width of the pulp chamber in 473 maxillary and 429 mandibular first molars on the radiographs and its reduction in size from the age of 11-14 years (19). On the other hand, Chandler *et al. *found significant differences in four aspects of pulp spaces between genders and suggested that human first molar pulps exhibit sexual dimorphism (9). Based on the results of this study, in presence of age factor, restoration had not significant effect on the mean of pulp chamber dimensions, aging affected these significantly in presence of restoration; so, it could be concluded that aging is the principal factor that cause changing in pulp chamber dimensions.

Oi *et al. *(20) who used micro-computed tomography (CT) for three dimensional observations of pulp cavities in the maxillary first premolar teeth found that mesial- distal widths and heights of the pulp cavity decreased with age.

According to Deutsch *et al. *(21), distance from the cusp tips to the furcation and the ceiling of the pulp chamber were the least variable measurements for bicuspids. Indeed, Deutsch *et al. *(21,22) as well as Kranser and Rankow (23) suggested that this consistency of cusp tip to ceiling and furcation gives an especially important finding for developing an access technique without perforation. In our study however, this consistency has only been approved for mean distance between cusp tip and furcation in both maxillary and mandibular first molars (Measuring dimension I).

Reported measurements in this study and their similarity to measurements in other studies (14,21,22) may give a general guideline for a more quantitative approach to first molar access cavity preparation in young as well as elders. Based on the results of present study, (Table 1) and (Table 2) the distance from cusp tip to furcation in maxillary first molar in both age groups was approximately 1mm more than mandibular first molar.

## CONCLUSION

The present study has shown that the dimensions of the pulp chambers in maxillary and mandibular first molars decrease with age; and in presence of age factor, restoration had not significant effect on the mean of pulp chamber dimensions. This finding may enhance knowledge to minimize errors during endodontics treatments.
